# Improving Pediatric Ovarian Torsion Evaluation in the Pediatric Emergency Department: A Quality Improvement Initiative

**DOI:** 10.1097/pq9.0000000000000709

**Published:** 2023-12-12

**Authors:** Brian L. Park, Sara Fenstermacher, A. Luana Stanescu, Lori Rutman, Lauren Kinneman, Patrick Solari

**Affiliations:** From the * Department of Pediatrics, Division of Emergency Medicine, Seattle Children’s Hospital, University of Washington School of Medicine, Seattle, CA, USA; † Department of Radiology, Seattle Children’s Hospital, University of Washington School of Medicine, Seattle, CA, USA.

## Abstract

**Background:**

Transabdominal pelvic ultrasound (TPUS) is the diagnostic test of choice for the evaluation of ovarian torsion, a time-sensitive surgical emergency. A full bladder is required to visualize the ovaries. Bladder filling is a time-consuming process leading to delays to TPUS, poor visualization of ovaries requiring repeat studies, and prolonged emergency department length of stay (ED LOS). The primary objective was to decrease the time to TPUS by standardizing the bladder filling process.

**Methods:**

This quality improvement initiative occurred at a single, academic, quaternary-care children’s hospital ED and utilized the Institute for Healthcare Improvement Model for Improvement with sequential plan-do-study-act cycles. The first set of interventions implemented in August 2021 included a new electronic order set and bladder scan by ED nurses. Subsequent plan-do-study-act cycles aimed to decrease the time to intravenous fluid, decrease fluid requirement, and decrease the need for intravenous fluid. The primary outcome measure was the monthly mean time to TPUS. Secondary outcome measures included monthly mean ED LOS and percentage of repeat TPUS. We performed data analysis with statistical process control charts to assess for system change over time.

**Results:**

The preintervention baseline included 292 ED encounters more than 10 months, and postintervention analysis included 526 ED encounters more than 16 months. Time to TPUS decreased (138–120 min), ED LOS decreased (372–335 min), and repeat TPUS decreased (18% to 4%). All changes met the rules for special cause variation.

**Conclusions:**

Standardizing the bladder filling process was associated with decreased time to TPUS, ED LOS, and repeat TPUS.

## INTRODUCTION

### Problem Description

Abdominal pain is one of the most common presenting complaints in the pediatric emergency department (ED) with a broad differential diagnosis.^1^ An important consideration in children with ovaries is ovarian or adnexal torsion, a time-sensitive surgical emergency. Transabdominal pelvic ultrasound with Doppler (TPUS) is the diagnostic test of choice when evaluating ovarian torsion and other ovarian pathologies in children. It requires a full bladder as a sonographic window to adequately visualize the ovaries.^2^ Patients presenting with significant abdominal pain for a surgical emergency often have an underfilled bladder due to accompanying nausea, vomiting, and anorexia.^3^ Before implementing our quality improvement (QI) initiative, the bladder filling process in our ED varied by provider. This variability resulted in long wait times to TPUS, prolonged ED length of stay (ED LOS), and repeat imaging studies due to inadequate visualization of the ovaries.

Ovarian torsion occurs when an ovary rotates on its ligamentous supports, leading to partial or complete obstruction of its blood flow.^4^ In addition to causing significant pain, prolonged torsion can lead to necrosis of the affected ovary and potentially affect fertility.^2^ Diagnosis of ovarian torsion is challenging due to its nonspecific clinical presentation and limitations of available diagnostic tests. Although TPUS has relatively low sensitivity (43%–70%), it is currently the first-line diagnostic test for children.^5–7^ A patient’s subjective bladder fullness, while easy to obtain, is often inaccurate.^8^ In addition to its use as a bedside diagnostic tool in the ED, point-of-care US evaluations of the bladder have been shown to decrease time to TPUS and increase first-time success in the visualization of the ovaries in other pediatric ED settings.^8–11^ In addition, a pilot randomized control trial comparing a novel rapid infusion device (LifeFLow; 410 Medical, Durham, N.C.) to normal intravenous (IV) fluid boluses demonstrated that rapid infusion fluids led to earlier bladder fullness and decreased time to TPUS in children.^12^

### Rationale

Given the unique requirement of a full bladder for TPUS compared with other abdominal sonographic studies, an important rate-limiting step for TPUS in the pediatric ED is bladder filling. We identified two areas of variability in our ED using a process map—assessment of bladder fullness and filling.

**Fig. 1.**
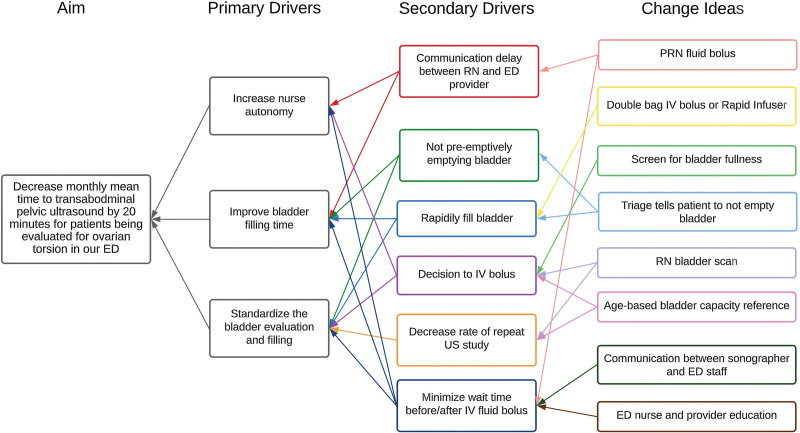
Key driver diagram. RN, registered nurse.

We hypothesized that objectively measuring the patient’s bladder volume using US would enable the team to determine when the patient was ready for a successful TPUS evaluation, thereby decreasing the frequency of repeat studies. This approach, in turn, would presumably decrease the overall ED LOS. Reducing variability in delivery of care may result in better outcomes in time-sensitive surgical emergencies.^13^ Figure 1 shows the key driver diagram demonstrating our theory of improvement.

### Specific Aims

The primary aim of this QI initiative was to decrease the time to TPUS by 20% through standardization of the bladder filling process for patients being evaluated for ovarian pathologies in our ED more than 1 year. Secondary outcomes included ED LOS and percent of repeat TPUS due to inadequate visualization of the ovaries.

## METHODS

The institutional review board of the Seattle Children’s Hospital/University of Washington exempted this study as QI and not human research.

### Context

The study occurred in the pediatric ED at a free-standing, quaternary-care, academic children’s hospital with more than 50,000 annual visits. Residents, medical students, and advance practice professionals provide patient care under the supervision of pediatric emergency medicine fellows and attending physicians. The emergency medicine division at our institution engages in multiple research and QI initiatives. The ED team is accustomed to integrating new care processes and generally accepts changes in the clinical environment. Additionally, the ED has standardized clinical pathways for common pediatric conditions with an accompanying electronic medical record (EMR) (Epic Systems Corporation, Verona, Wis.) order set, which bundles diagnostic evaluation (eg, laboratory and imaging studies) and interventions such as IV fluids and antibiotics.

### Interventions

Implementation of the new ovarian torsion evaluation process occurred on August 19, 2021, with the “ED Ovarian Torsion” order set update. The QI team announced the new clinical pathway and order set at the ED physician and nursing division meetings and through an electronic newsletter for the department. The updated order set included routine bladder scanning (Portascan 3D; Laborie, Orangeburg, N.Y.) by the patient’s nurse on initiating the order set by the patient’s physician or APP when there is clinical concern for an ovarian pathology requiring TPUS. The order set includes a minimum bladder volume threshold to reach before TPUS based on age (>300 mL for 8 to <14 y of age; >350 mL for ages 14 y of age or older). We determined the bladder volume threshold using an age-based equation and simplified it with the radiology team’s input for ease of use.^14^ If the patient’s bladder volume was below the determined threshold, the nurse initiated an IV fluid bolus with 0.9% sodium chloride at 20 mL/kg (maximum 1,000 mL). The nurse can initiate up to two fluid boluses at 20 mL/kg each as *pro re nata* to streamline the process. The patient’s nurse delivered IV fluid through a single IV pump (Becton Dickinson, Franklin Lakes, N.J.) at 999 mL/h for fluid volume ≤500 mL or through two IV pumps at these settings combined into a single IV to deliver up to 1,999 mL/h for fluid volumes >500 mL. The patient’s physician or APP could also use a rapid infuser device (Belmont Medical Technologies, Billerica, Mass.) to deliver fluid at a maximum of 750 mL/min. The QI team encouraged using the rapid infuser but did not mandate it due to only one rapid infuser available in our ED. The patient’s nurse then scanned the patient’s bladder every 30 minutes until the bladder volume reached the threshold. At this point, the patient’s nurse contacts the US technologist to initiate TPUS. The QI team left the use of the order set up to the provider’s discretion. Still, it recommended its use for “healthy” children without underlying malignancy, inflammatory bowel disease, or renal or cardiac disease.

**Fig. 2.**
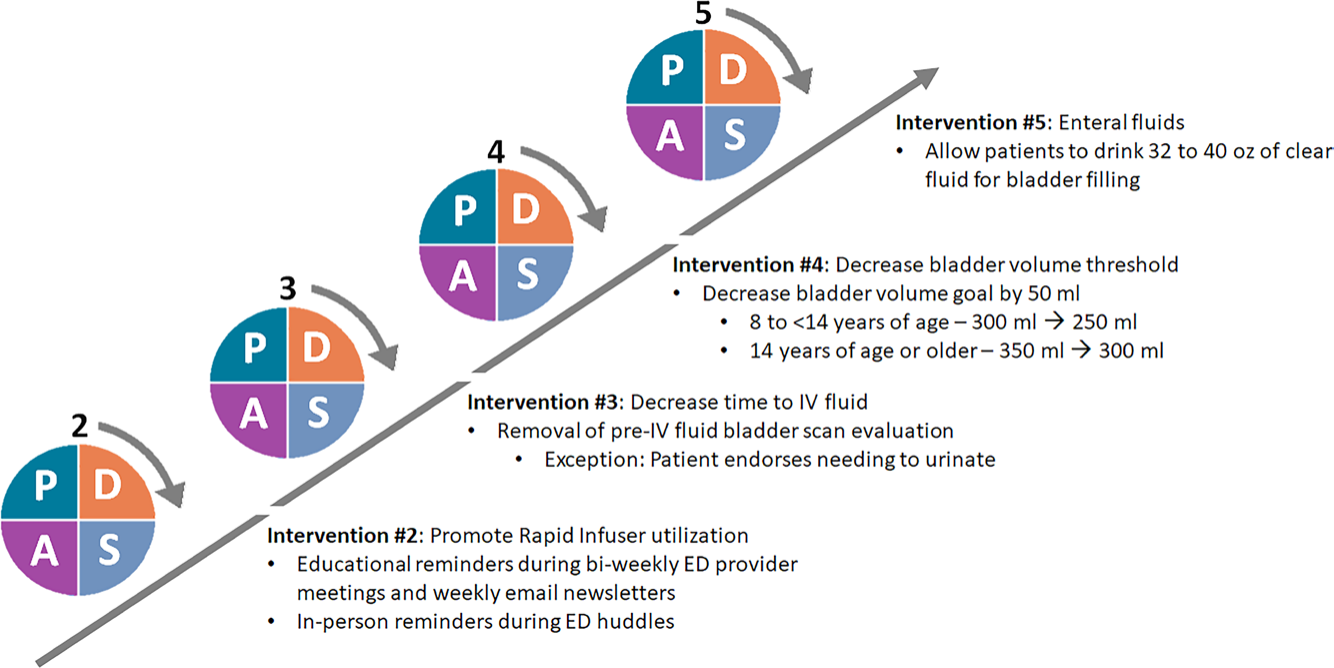
PDSA ramp: subsequent interventions. PDSA, plan do study act.

Figure 2 shows the plan-do-study-act ramp with subsequent interventions. The second intervention focused on improving the utilization of the rapid infuser through electronic newsletter reminders and announcements during biweekly ED division meetings with accompanying education on the safety and utility of the rapid infuser. The third intervention removed the routine bladder scan before the first fluid bolus while continuing the bladder scan after fluid boluses, as most patients required IV fluids before TPUS evaluation. The fourth intervention decreased the minimum bladder volume thresholds (>250 mL for 8 to <14 y of age; >300 mL for 14 y of age or older) to decrease fluid requirement. The fifth and final intervention was the introduction of enteral fluids. In response to the ED volume surge during this period with longer than usual waiting times, we gave patients 32 to 40 oz of clear fluid (eg, water, electrolyte drinks, soda, etc.) to drink (if able to tolerate) while in the waiting room after they underwent a rapid medical evaluation by an attending ED physician and had clinical presentation concerning for ovarian torsion. We also offered enteral fluids for roomed patients awaiting IV placement and fluid bolus. Giving enteral fluids was up to the discretion of the physician evaluating the patient.

### Measures

The primary outcome measure of this QI project was the monthly mean time to TPUS. This measured the time from the placement of the imaging order to the initiation of the imaging study. These two-time points are automatically generated in the EMR when the patient’s provider places the imaging order and when the US technologists initiate the imaging study. The secondary outcome measures were ED LOS and the percent of repeat imaging due to inadequate visualization. ED LOS is measured from when an ED provider (eg, attending physician, fellow, resident, or medical student) assigns themselves to the patient in the EMR to when the patient is marked ready to be discharged in the EMR. The start time for (purpose of calculating ED LOS) for those who underwent rapid medical evaluation in the waiting room began once they were roomed in the ED, and an ED physician assigned themselves to the patient. The QI team determined the percentage of repeat imaging through a documentation review in the EMR.

The process measures included the percentage of order panel utilization when obtaining TPUS and the time to the first IV bolus. We distinguished utilization of the order panel from ordering TPUS outside the order set through a manual review of the patient’s EMR. The QI team calculated the time to first IV fluid based on the documented time of IV fluid administration by the nurse and when the provider placed the IV fluid order.

The balancing measure included the percentage of patients requiring IV fluid usage. This measure was of interest as we suspected that routine administration of IV fluids might lead to increased IV fluid utilization. Although a full bladder helps the sonographers better visualize the ovaries, an underfilled bladder does not necessarily lead to an inadequate study. Before implementing the minimum bladder volume threshold, some patients likely had an adequate TPUS without having a full bladder.

### Data Collection and Analysis

To obtain baseline data, the QI team identified all ED encounters with TPUS evaluation ten months prior (October 1, 2020, to October 31, 2021) to the implementation of QI interventions. We chose this time frame as it coincides with the introduction of a new EMR system in our ED. The QI team manually reviewed each encounter to verify patient demographics, inclusion/exclusion criteria, time metrics of interest, instances of repeat USs, fluid administration, and operative diagnosis. We collected postintervention data for 19 months. We included any patients with ovaries (ie, 8 y of age and older) undergoing TPUS to evaluate ovarian pathologies. We excluded patients with malignancy, shock, inflammatory bowel disease, or underlying renal or cardiac disease.

We utilized statistical process control to assess change in the measures of interest over time. We used X-bar and S charts for continuous-time variables (eg, time to TPUS and ED LOS) and P charts for changes in proportions (eg, repeat TPUS and order set utilization), with monthly subgroups due to the relatively low frequency of these studies. Data were analyzed on Excel (Microsoft Corporation, Redmond, Wash.) and QI-Charts Add-in for Excel (Process Improvement Products, Austin, Tex.).

### Results

Nine hundred sixty-eight ED encounters met the inclusion criteria, with 31 encounters excluded per criteria during the study period. Of the 937 encounters included for analysis, 304 occurred during the preimplementation phase, and 633 occurred during the postimplementation phase. Table 1 shows patient demographic and operative diagnoses.

**Table 1.** Pre-/Postimplementation Patient Demographics

	Pre, n = 304	Post, n = 633	Total, n = 937
Age in years (SD)	13.8 (2.8)	13.9 (2.7)	13.9 (2.8)
Race			
White	173 (56.9%)	315 (49.8%)	488 (52.1%)
Black or African American	30 (9.9%)	54 (8.5%)	84 (9.0%)
Asian	15 (4.9%)	43 (6.8%)	58 (6.2%)
American Indian, Alaska Native, Native Hawaiian, and other Pacific Islander	0 (0%)	2 (0.3%)	2 (0.2%)
2 or more races	7 (2.3%)	36 (5.7%)	43 (4.6%
Other	59 (19.4%)	116 (18.3%)	175 (18.7%)
None provided	20 (6.6%)	67 (10.6%)	87 (9.3%)
Ethnicity			
Hispanic	62 (20.4%)	165 (26.1%)	227 (24.2%)
Non-Hispanic	229 (75.3%)	410 (64.8)	639 (68.2%)
None provided	13 (4.3%)	58 (9.2%)	71 (7.6%)
Ovarian torsion	16	16	32
No mass/cyst	5	8	13
Mass/cyst	11	8	19

**Fig. 3.**
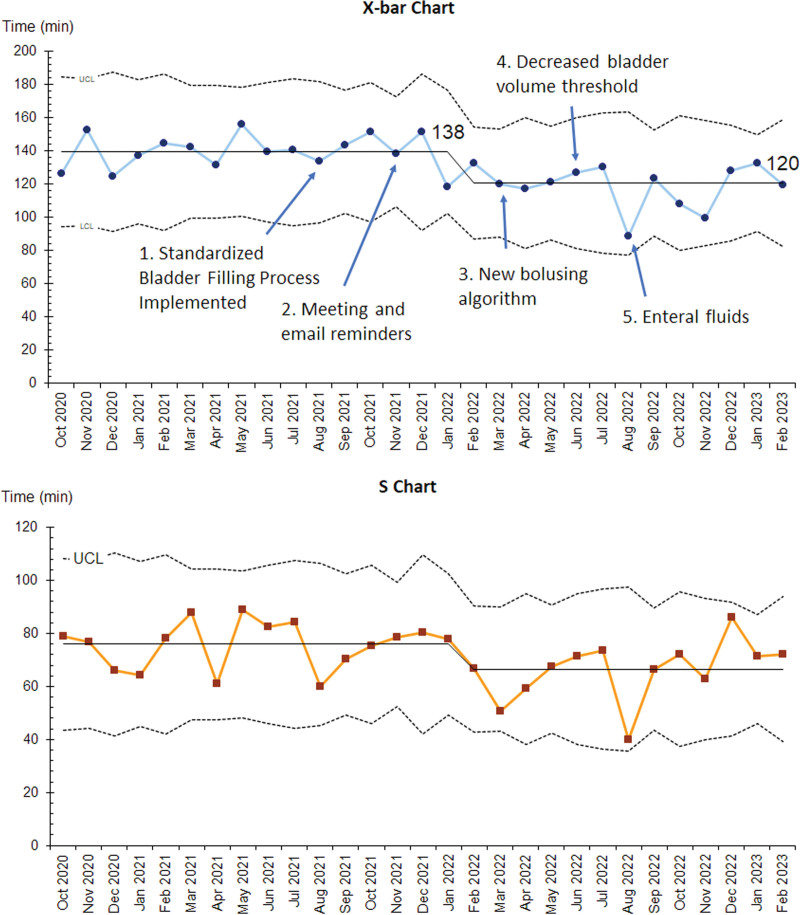
Monthly mean time to transabdominal pelvic US (X-bar and S chart). LCL, lower control limit; UCL, upper control limit.

**Fig. 4.**
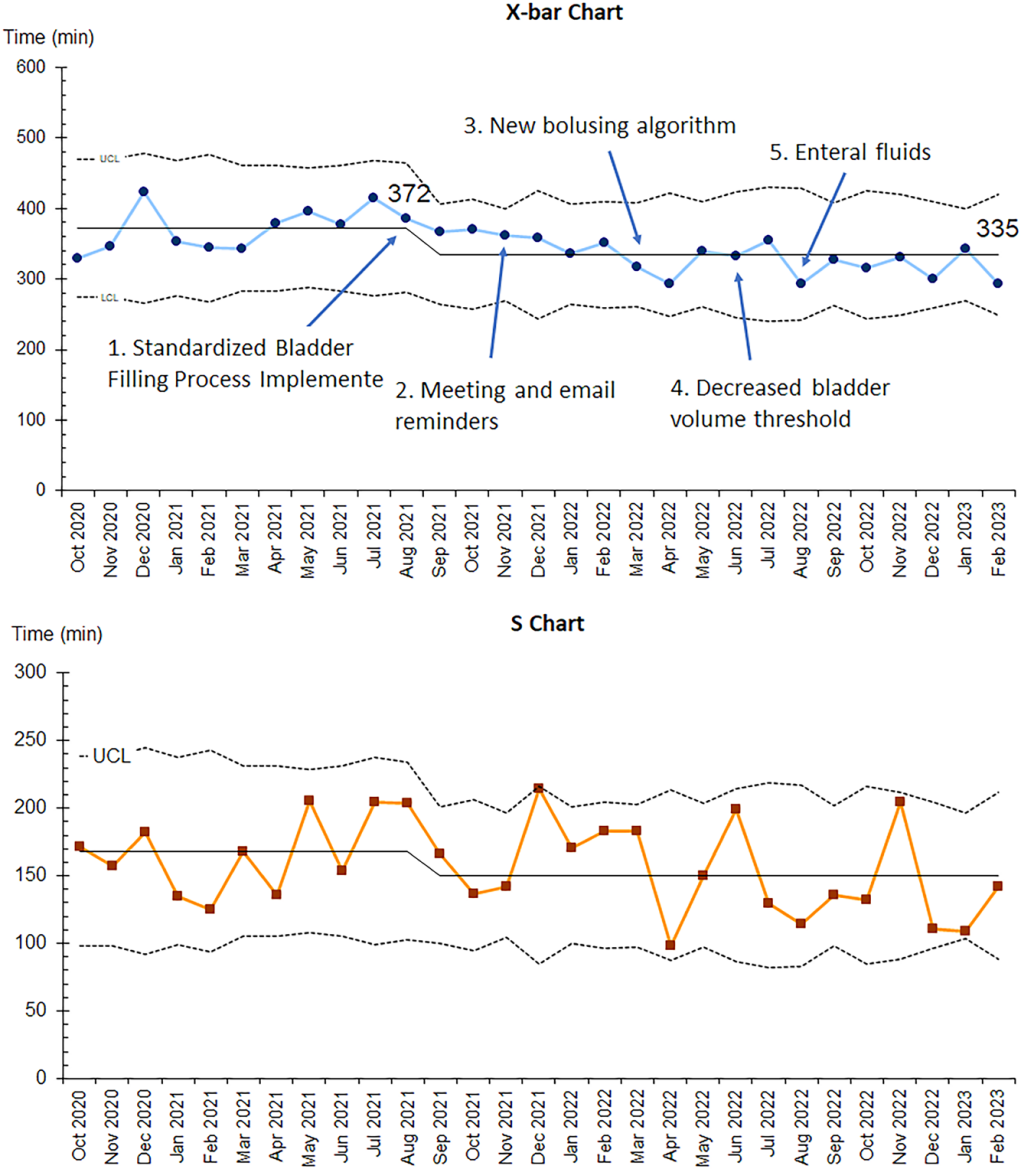
Monthly mean ED LOS (X-bar and S chart). LCL, lower control limit; UCL, upper control limit.

**Fig. 5.**
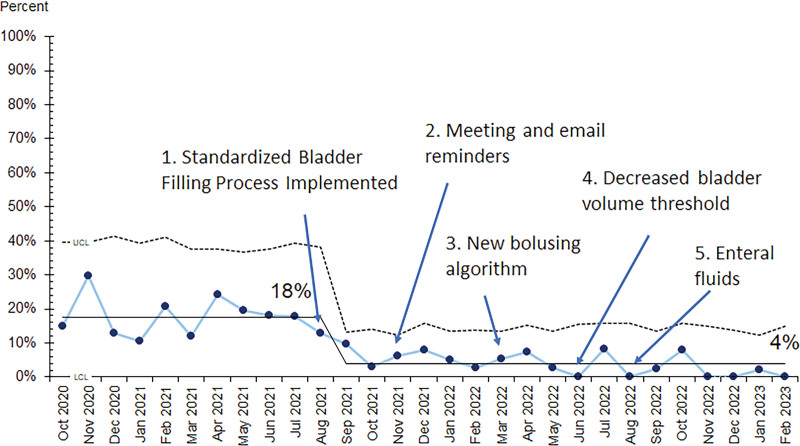
Proportion of repeat US due to inadequate initial study (P chart).

All three outcome measures demonstrated improvement with special cause variation. The monthly mean time to TPUS decreased from 138 to 120 minutes starting in January 2022 (Fig. 3). The monthly mean ED LOS decreased from 371 to 335 minutes, and the rate of repeat TPUS decreased from 18% to 4% with the implementation of the first set of interventions in August 2021 (Figs. 4 and 5).

Order panel utilization increased from a low of 44% to a high of 90% over the first 6 months and a mean of 66% in the postimplementation period. The mean percentage of patients receiving IV fluid remained stable at 83% until the introduction of enteral fluids (fourth intervention, August 2022), with a decrease to 60% with special cause variation (**Supplemental Digital Content 1**, which describes proportion of patients receiving IV fluids (P chart), http://links.lww.com/PQ9/A531). Monthly mean time to IV fluid remained at 43 minutes without any changes meeting special cause variation (**Supplemental Digital Content 2**, which describes monthly mean time to IV bolus (X-bar and S chart), http://links.lww.com/PQ9/A532). We observed low adoption of the rapid infuser, with only three instances of its use during the first 6 months postimplementation. We also observed a significant increase in our monthly ED volume starting around the time of the initial interventions, with a peak volume of 3,928 during preimplementation in July 2021 compared with the peak of 7,549 in November 2022 (**Supplemental Digital Content 3**, which describes monthly ED volume versus mean time to transabdominal pelvic US, http://links.lww.com/PQ9/A533).

## DISCUSSION

Combining bladder scanning and nurse-initiated IV fluid administration, the initial set of interventions aimed to decrease delays to bladder filling. The mean time to TPUS decreased with sustained improvement several months into the postimplementation period; however, it did not improve further with subsequent interventions. The percentage of repeat TPUS improved with the initial set of interventions and appeared to coincide with the decrease in ED LOS. Conversely, the addition of routine bladder scanning likely added time to TPUS while improving the first-time success of the study.

Other studies have assessed the utility of point-of-care US by ED physicians for bladder evaluation before TPUS.^8,9^ Although it is a common ED nursing procedure, a nurse performed bladder scanning in this context has not been reported in the literature. In addition to this difference, these prior prospective studies used the bladder shape^8^ and the height of the bladder relative to the uterus.^9^ We used numerical bladder measurements to minimize subjectivity and ease of use (bladder scanner automatically calculates bladder volume). Furthermore, this allowed for easy incorporation into the nursing workflow without requiring additional training.

There were several challenges during the postimplementation period. A major hurdle was the increase in ED patient volume during this period with the reversal of pandemic-related social distancing guidelines and children returning to in-person classes—a shared experience with many other pediatric EDs. Monthly ED visits have steadily increased since implementing interventions and, at times, surpassed even prepandemic ED patient volumes. The significant ED nursing and sonographer turnover compounded this issue, as the interventions largely depended on nurses and US imaging. Although it is difficult to quantify their effects, the increasing patient volume combined with high staff turnover likely hurt the outcome measures. A third challenge was the poor adoption of the rapid infuser to deliver IV fluids. Some of the concerns were the availability of the rapid infuser for emergencies, as there is only one device in the ED and the cost to the patient. Despite educational sessions during department meetings and frequent reminders, utilization of the rapid infuser remained low.

Implementing enteral fluids as part of the bladder-filling process was in response to the increasing ED volume and waiting room times. Having patients drink clear fluids while in the waiting room or waiting for IV placement decreased IV fluid usage without negatively impacting the outcome measures. The observed change also suggests that some patients declined IV fluid entirely and filled their bladders by drinking (typical for nonemergent, outpatient TPUS) without causing a delay.

The time to IV fluids was longer than anticipated and constituted a third of the time for TPUS evaluation. Removal of bladder scan before administering fluid (with an exception as shown in Fig. 2) was to decrease this time as preceding months demonstrated that most patients required fluids. This intervention, however, did not affect the time to IV fluids and may be partly attributable to increasing patient volume and nurse staffing challenges. Additionally, the use of enteral fluid potentially mitigates prolonged time to IV fluids by avoiding IV fluids altogether.

An interesting finding was the same incidence of ovarian torsions in the pre- and postimplementation periods despite the higher number of patients and longer study duration in the postimplementation period. These cases only reflect ovarian torsion diagnosed with TPUS in our ED and do not include patients diagnosed at other institutions and transferred to our ED for surgical management. Additionally, the higher number of TPUS performed likely reflects the increase in visits to the ED, as abdominal pain is a common chief complaint. It is important to recognize that although ovarian torsion is relatively rare due to its potential for serious complications with a missed or delayed diagnosis and nonspecific clinical presentation, evaluation with TPUS is often indicated in children with ovaries presenting with abdominal pain. Thus, improvement to this time and resource-consuming process may improve overall ED throughput.

### Limitations

There are several limitations to this study. First, the variability in the clinical environment due to significant patient volume increase and critical staffing turnover during the postimplementation period makes it challenging to assess the true magnitude of the interventions. However, it is unlikely that these environmental changes would have positively affected the outcome measures. Additionally, given the relatively high rates of repeat TPUS before the preimplementation period, the calculated time to TPUS during this phase likely skewed toward a lower time as the administrative data used does not create a second timestamp when a patient returns for a second TPUS after an inadequate first study. Due to this, it is possible that not all instances of repeat TPUS were properly documented and captured in the data; however, it is unlikely that this alone would explain the observed improvement. Second, the use of oral fluids as part of the bladder filling process in evaluating a surgical emergency may be controversial due to preoperative *nil per os* timing. Although our institution’s anesthesiology department approved this approach as it would likely not delay surgical intervention, this may not be generalizable in other pediatric EDs. Additionally, *nil per os* timing does not apply to children undergoing emergency surgery at our institution (and presumably in other institutions). Third, we did not assess the utility of transvaginal pelvic US—a study requiring no bladder filling. As a more invasive study compared to TPUS, we limit transvaginal USs to sexually active patients with their consent, and, in practice, we rarely use this study. In a general ED serving an older population, transvaginal USs may be an option.

### Concluding Summary

Standardizing the bladder filling process for TPUS evaluation of ovarian torsion and other ovarian pathologies led to a decrease in time to TPUS, ED LOS, and frequency of repeat TPUS due to inadequate visualization of the ovaries. Contrary to our expectations, this did not lead to an increase in IV fluid utilization. Additionally, oral fluids may be a good adjunct or an alternative to IV fluids for bladder filling in the pediatric ED; however, this practice may be controversial and not generalizable to other pediatric EDs.

## Supplementary Material


