# Supplementary figures and images for: Improving Pediatric Ovarian Torsion Evaluation in the Pediatric Emergency Department: A Quality Improvement Initiative
Source: Pediatr Qual Saf. 2023 Dec 12;8(6):e709. doi: 10.1097/pq9.0000000000000709 (PMC10715784; doi:10.1097/pq9.0000000000000709)

Percent

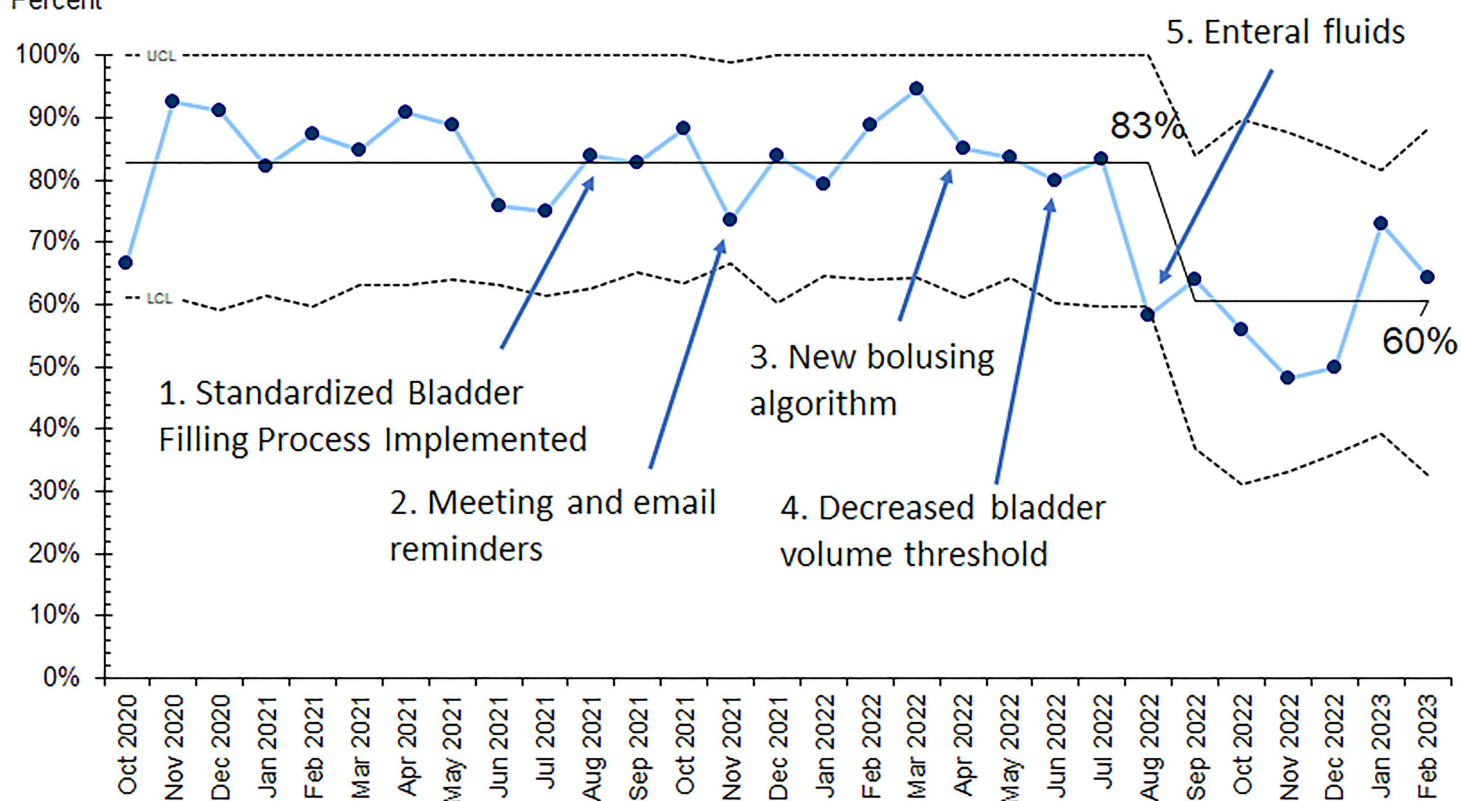

Supplement: Supplementary file 1 [file pqs-8-e709-s001.pdf]

**X-bar Chart**

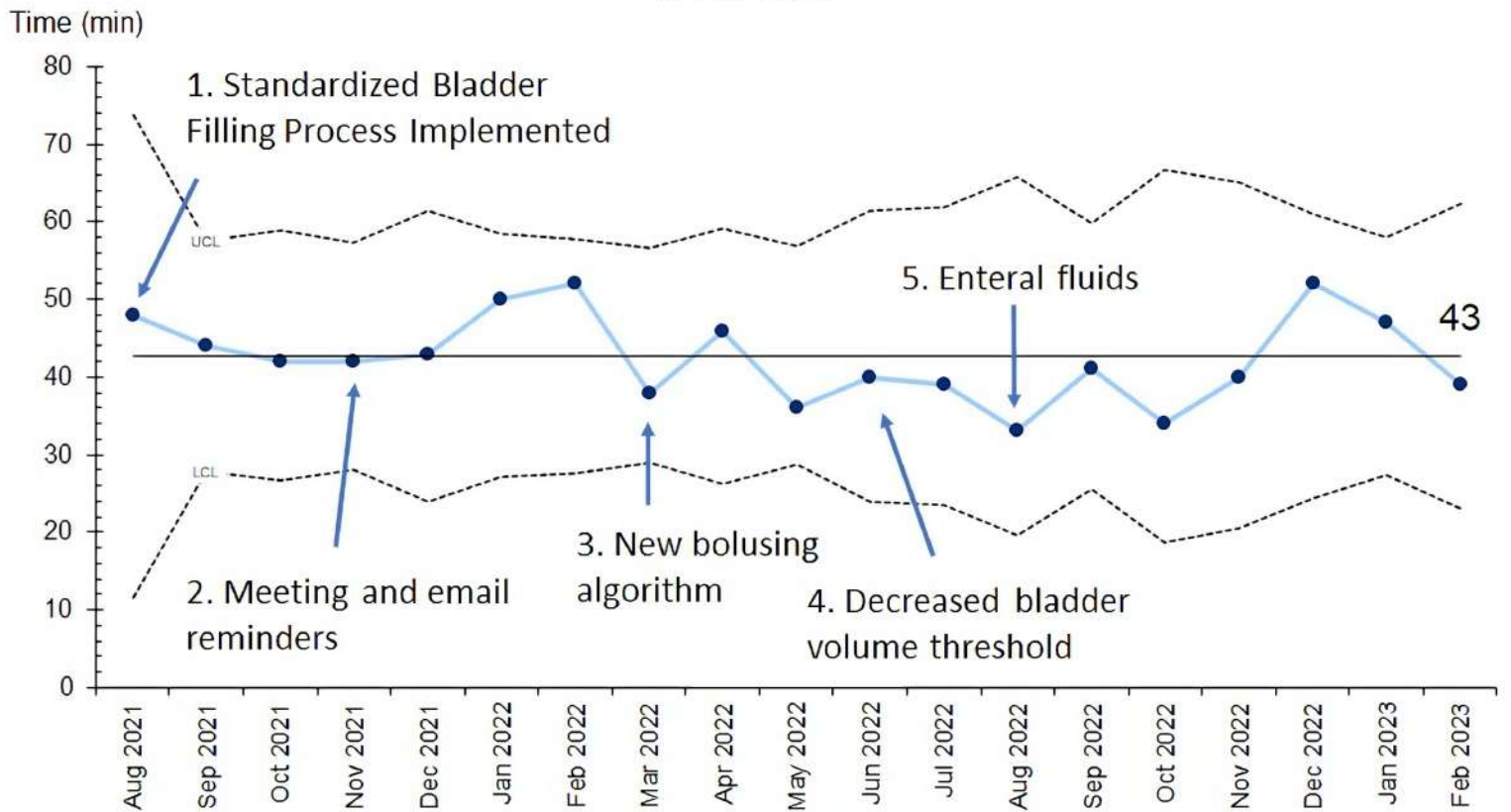

**S Chart**

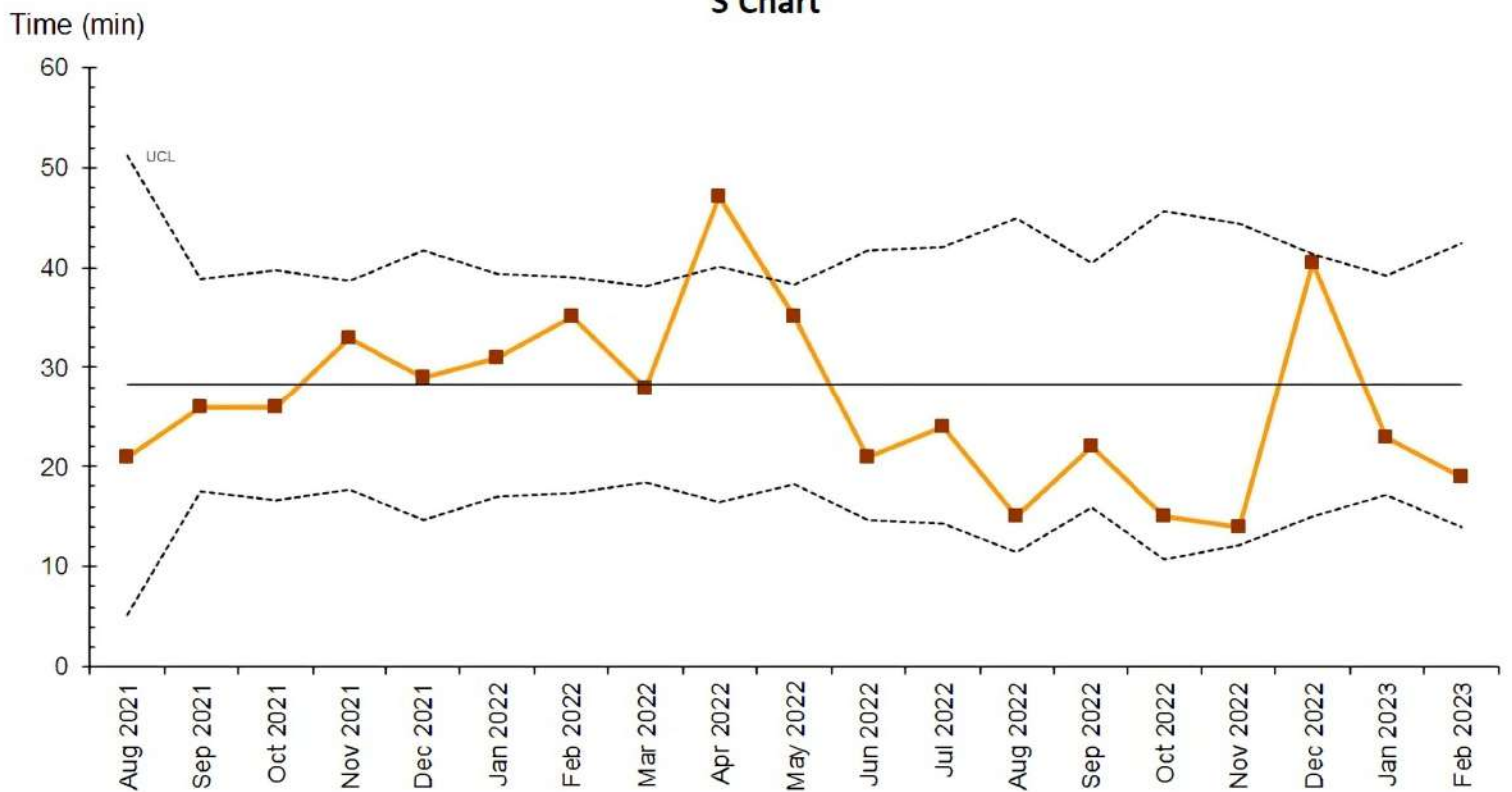

Supplement: Supplementary file 2 [file pqs-8-e709-s002.pdf]

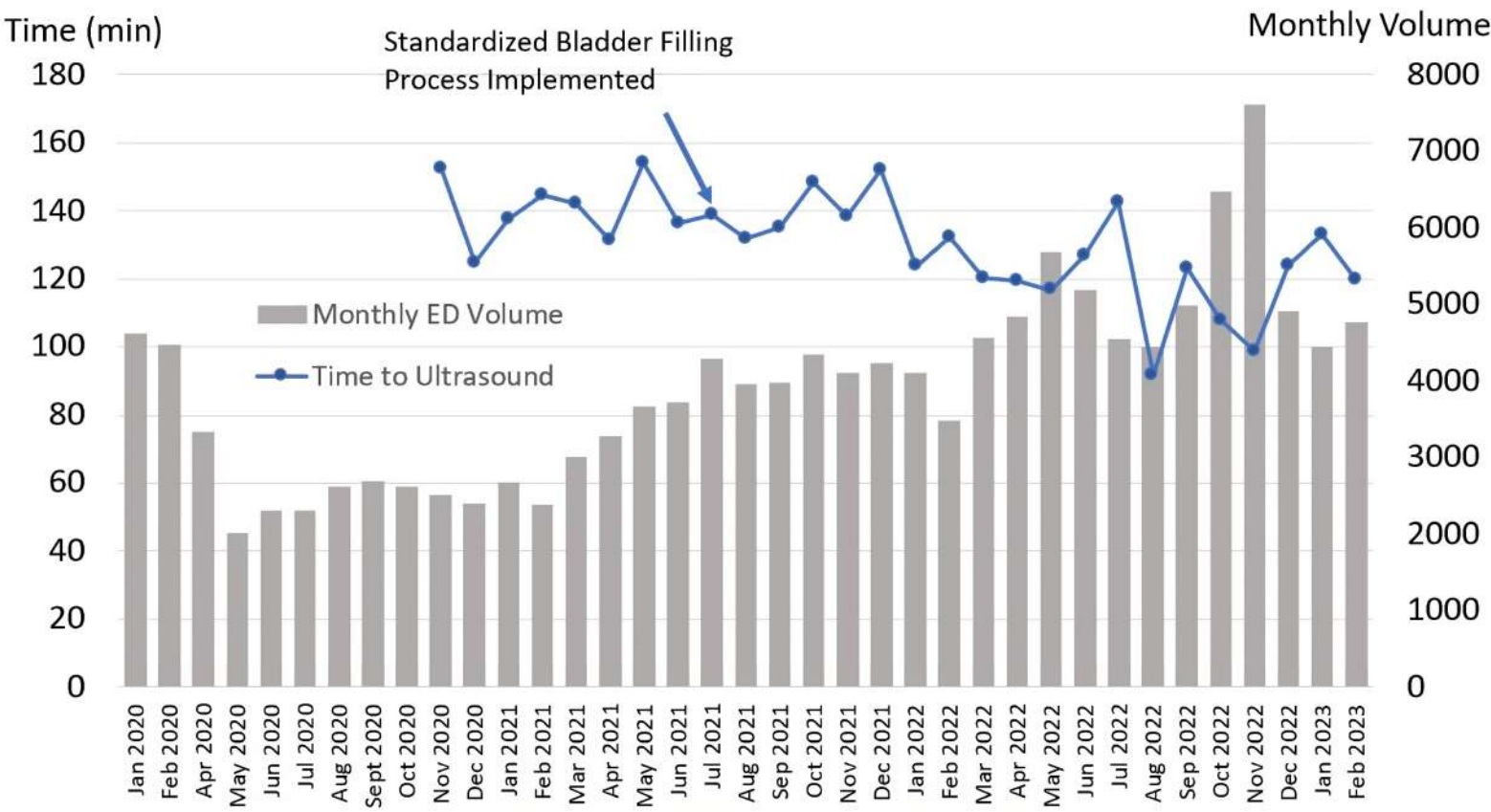

Supplement: Supplementary file 3 [file pqs-8-e709-s003.pdf]
